# Whole Brain Magnetic Resonance Image Atlases: A Systematic Review of Existing Atlases and Caveats for Use in Population Imaging

**DOI:** 10.3389/fninf.2017.00001

**Published:** 2017-01-19

**Authors:** David Alexander Dickie, Susan D. Shenkin, Devasuda Anblagan, Juyoung Lee, Manuel Blesa Cabez, David Rodriguez, James P. Boardman, Adam Waldman, Dominic E. Job, Joanna M. Wardlaw

**Affiliations:** ^1^Brain Research Imaging Centre, Neuroimaging Sciences, Centre for Clinical Brain Sciences, Royal Infirmary of Edinburgh, The University of EdinburghEdinburgh, UK; ^2^Scottish Imaging Network, A Platform for Scientific Excellence (SINAPSE) CollaborationGlasgow, UK; ^3^Geriatric Medicine Unit, Royal Infirmary of Edinburgh, The University of EdinburghEdinburgh, UK; ^4^Department of Psychology, Centre for Cognitive Ageing and Cognitive Epidemiology, The University of EdinburghEdinburgh, UK; ^5^MRC Centre for Reproductive Health, Queen's Medical Research InstituteEdinburgh, UK; ^6^Graduate Training Centre of Neuroscience, International Max Planck Research School, University of TübingenTübingen, Germany

**Keywords:** brain mapping, MRI imaging, atlases as topic, brain, systematic review, aging, neurodevelopment, neurodegeneration

## Abstract

Brain MRI atlases may be used to characterize brain structural changes across the life course. Atlases have important applications in research, e.g., as registration and segmentation targets to underpin image analysis in population imaging studies, and potentially in future in clinical practice, e.g., as templates for identifying brain structural changes out with normal limits, and increasingly for use in surgical planning. However, there are several caveats and limitations which must be considered before successfully applying brain MRI atlases to research and clinical problems. For example, the influential Talairach and Tournoux atlas was derived from a single fixed cadaveric brain from an elderly female with limited clinical information, yet is the basis of many modern atlases and is often used to report locations of functional activation. We systematically review currently available whole brain structural MRI atlases with particular reference to the implications for population imaging through to emerging clinical practice. We found 66 whole brain structural MRI atlases world-wide. The vast majority were based on T1, T2, and/or proton density (PD) structural sequences, had been derived using parametric statistics (inappropriate for brain volume distributions), had limited supporting clinical or cognitive data, and included few younger (>5 and <18 years) or older (>60 years) subjects. To successfully characterize brain structural features and their changes across different stages of life, we conclude that whole brain structural MRI atlases should include: more subjects at the upper and lower extremes of age; additional structural sequences, including fluid attenuation inversion recovery (FLAIR) and T2^*^ sequences; a range of appropriate statistics, e.g., rank-based or non-parametric; and detailed cognitive and clinical profiles of the included subjects in order to increase the relevance and utility of these atlases.

## Introduction

Structural magnetic resonance imaging (MRI) brain atlases, frequently also referred to in the literature as templates, are important tools for research and, increasingly, clinical practice. Individual brain scans from several individuals can be combined to form a brain image bank, which can in turn be used to form a brain atlas—an anatomical representation of the brain showing group-wise or study population global or regional brain features.

The terms “brain atlas” and “brain template” have both been used commonly in the literature to date; while they may have different meanings in some situations, many papers do not make this clear but rather appear to use the terms interchangeably. Therefore, for the interests of this paper, we focus on using the term “atlas” but use both terms interchangeably. Atlases are derived by statistically summarizing, e.g., averaging, voxel-wise, regional, or global brain MRI measures from several individuals and they may be used in research as registration targets for functional activation, segmentation, and statistical mapping, for example in analysis of population imaging datasets (Good et al., 2001; Buckner et al., 2004; Avants et al., 2008). In the future, atlases may also be used in clinical practice as reference images to support diagnoses of age-related neurodegenerative disorders (Farrell et al., 2009); therefore their reliability and relevance to the clinical population on which they are being used is paramount.

Brain structure in old age and early life is different to brain structure in younger and middle-aged adults (Gur et al., 1991; Courchesne et al., 2000; Good et al., 2001; Sowell et al., 2003). For example, the developing brain presents specific challenges to atlas construction because of marked variations in head size and shape in early life, maturational processes leading to changes in signal intensity profiles (for example, reducing brain water content and increasing cell density over the perinatal period), relatively lower spatial resolution (cortical patterning at term birth is broadly similar to adult patterns but is approximately one third of the volume at adulthood), and lower contrast between tissue classes (Matsuzawa et al., 2001). In children >5 years, the brain is still developing at an accelerated rate. These issues invalidate the application of adult atlases to data acquired during development, because of misclassification of tissues and structures (Muzik et al., 2000; Yoon et al., 2009), and have led to the development of age-specific atlases for early life studies.

In older age the ventricles, particularly the lateral ventricles, and sulci spaces are generally larger, the gray matter and white matter atrophy in varying proportions, and white matter hyperintensities (WMH) are often present (Lemaitre et al., 2005; Dickie et al., 2015b, 2016b). These and the other many features of brain aging, e.g., lacunes, microbleeds and enlarged perivascular spaces, require specific T2-based sequences, such as fluid attenuated inversion recovery (FLAIR) and T2^*^, to be captured effectively (Wardlaw et al., 2013). Because of these differences in brain structure, the use of an atlas based on only younger subjects and a limited range of sequences can create a bias in life course population studies, e.g., systematic overexpansion (Buckner et al., 2004) or regional distortion of older brains. Even within restricted age bands brain structure is highly variable due to various factors such as ethnicity, medical history, e.g., hypertension, smoking and cognition (Farrell et al., 2009; Wardlaw et al., 2011). Therefore, population brain atlases must include information on age, sex, ethnicity, relevant medical history, and cognitive testing to have broad uses and relevance. Further, brain atlases should be derived using statistical methods that effectively characterize the wide and irregular variance in brain structure across the life course (Dickie et al., 2013). Attempts to understand this variation and create brain atlases have increased exponentially with the advent of MR and other non-invasive imaging techniques but the origins of this pursuit extend back many thousands of years.

The gyral and sulcal pattern of the human brain is thought to have been first described in 3000 B.C. by Imhotep, an Egyptian “god” of medicine (Adelman and Smith, 1987). Although study of the structure of the brain continued for more than 4500 years, it was not until 1664 when Thomas Willis published *Cerebri Anatome* (“Anatomy of the Brain”) that robust methods for measuring brain structure started to be developed (O'connor, 2003). Willis directed novel autopsies of the brain in which it was first removed from the skull, in contrast to the traditional *in situ* dissections of the time, and then sliced from the base upwards. The slices were then viewed with a microscope and drawn by Christopher Wren (O'connor, 2003). These 350 year old drawings arguably represent the first attempt to create a brain atlas but more detailed atlases of the brains' cyto- and myelo-architecture did not emerge until the late nineteenth/early twentieth century (Betz, 1874; Brodmann, 1909, 1994; Von Economo and Koskinas, 1925). Such atlases are useful to understand the distribution of tissue types and fibers, but they have little use in modern clinical practice. One of the first clinically relevant atlases was published by Talairach et al. (1967), who developed a 3D coordinate system to assist deep-brain surgery.

The subsequent Talairach and Tournoux atlas (Talairach and Tournoux, 1988) has become one of the most influential atlases in brain imaging (Evans et al., 2012). This atlas provides a standardized set of coordinates to determine specific sites within the brain. It has been used to describe the site of a biopsy, or to compare data from structural MRI, functional MRI (fMRI), SPECT, and PET studies. However, the Talairach and Tournoux atlas has been described as “woefully inadequate” (Toga and Thompson, 2007). The reasons for this, including that it was derived from a single fixed cadaveric brain from an elderly female with limited clinical information, have been listed by many and well-known since the atlases' inception (Evans et al., 1993, 2012; Devlin and Poldrack, 2007). Indeed, they were noted in the original author's foreword, “this method is valid with precision only for the brain under consideration” (Talairach and Tournoux, 1988), but this may not be commonly known amongst users of this and derived atlases, e.g., Montreal Neurological Institute (MNI)152 (Brett et al., 2001). Population brain atlases, many of which were descended from Talairach (Evans et al., 2012), may therefore be lacking in age-appropriate, clinically, and cognitively described subjects that were synthesized via appropriate image analysis and statistical methods. It is for this reason that we undertook the following systematic review to identify, collate, and describe existing structural MRI brain atlases.

In this review, we aim to summarize the currently available structural MRI brain atlases across the life span—published in journals and/or on the internet—for researchers in population based imaging. Following our review we discuss the practical, technical, and statistical considerations that should be borne in mind when using brain image atlases.

## Materials and methods

We followed “Preferred reporting items for systematic reviews and meta-analyses (PRISMA)” reporting guidelines (Moher et al., 2009) in preparation of this manuscript. From October 2010 to April 2015, we systematically searched for “normal” brain structural MRI atlases. From April 2015 to August 2016, we supplemented this search with: hand searching of reference sections in previous review articles and records we included here (e.g., Mazziotta et al., 2001; Toga et al., 2006; Evans et al., 2012); periodical searching of Google with a subset of these terms; review of content alerts distributed by relevant journal articles, e.g., NeuroImage (http://www.journals.elsevier.com/neuroimage/), Human Brain Mapping [http://onlinelibrary.wiley.com/journal/10.1002/(ISSN)1097-0193], and Frontiers in Neuroscience (http://journal.frontiersin.org/journal/neuroscience); and, finally, hand searching of neuroimaging data sharing initiatives NeuroVault (http://neurovault.org/) and NITRC (http://www.nitrc.org/). Two authors (DAD and JYL) independently and systematically searched PubMed (including MEDLINE; http://www.ncbi.nlm.nih.gov/pubmed/), and the internet using Google (http://www.google.co.uk/) and Google Scholar (http://scholar.google.co.uk/) with the terms: “Magnetic Resonance Imaging” or “Magnetic Resonance Image” or “Magnetic Resonance Images” or “MRI” or “MR” and “brain” and “template” or “atlas” or “stereotactic” or “stereotaxic” and “human.”

October 2010-August 2016 was the time during which we conducted our search, there were no publication date restrictions on eligibility for inclusion and we included all normal MRI atlases of whole brain structures from across the lifespan. We included atlases with “anatomical” or “structural” sequences and probability maps, e.g., T1-, T2-, T2^*^-, FLAIR-weighted images, and gray matter (GM), white matter (WM), and cerebrospinal fluid (CSF) probability maps. We did not include atlases solely of segmented regional structures (ROI), such as subcortical GM or individual cortical areas (e.g., Westbury et al., 1999; Ahsan et al., 2007), or histological sections (e.g., Eickhoff et al., 2005), but did include atlases that had whole brain and regional structures. We excluded: (1) non-human brain atlases, e.g., macaque; (2) diffusion or functional MRI connectively atlases without anatomical/structural components, e.g., JHU ICBM-DTI-81 and NTU-90 (Yeh and Tseng, 2011); (3) functional MRI brain atlases only, e.g., http://www.brainmap.org/; (4) records that described atlas methods only (e.g., Maldjian et al., 2003; Wilke et al., 2008; Van Leemput, 2009; Chen et al., 2012); and (5) atlases that included patients with known neurological or central nervous system disease, e.g., Alzheimer's disease (Desikan et al., 2006; Loni, 2011).

We provide information reported in each structural MRI brain atlas on the number, age, and sex of participants; sequences collected; statistical derivation method; and clinical/cognitive data found.

## Results

**Figure 1:**
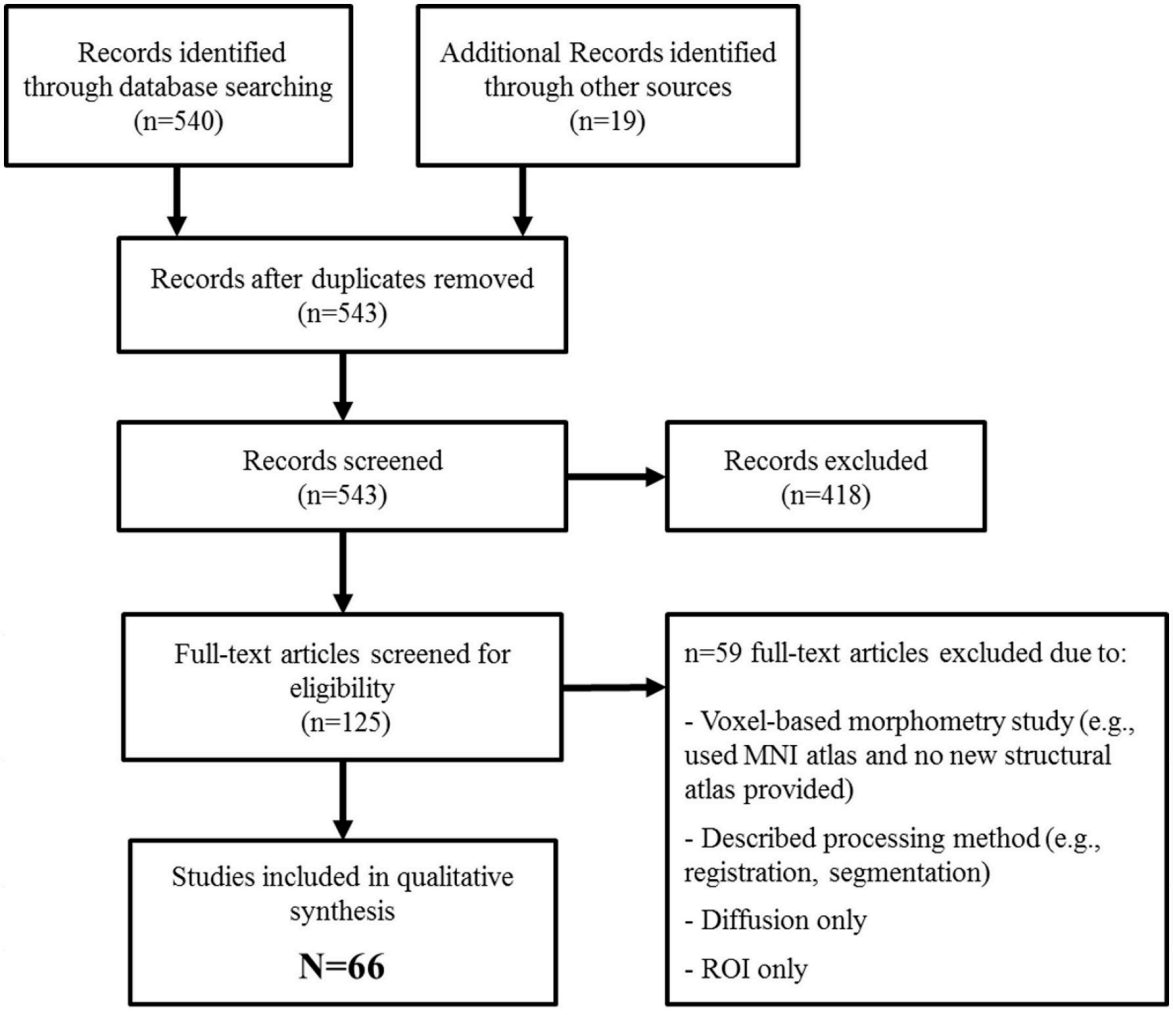
**PRISMA flow diagram of systematic identification of whole brain structural MRI atlases**.

We identified 543 potentially eligible records (Figure 1) of which 66 met inclusion criteria. Descriptions of each atlas are provided in Table 1.

**Table 1:** **Whole brain structural MRI atlases (alphabetical order by name)**.

**Name**	**Age^a^**	**N (Sex)**	**Sequences/contents**	**Derivation method**	**Clinical/cognitive data**
10–20 sensor placement system structural atlas (Kabdebon et al., 2014)	7.1 weeks	1 (*M* = 0; *F* = 1)	• T1 • T2 • Tissue maps • ROI	• Single subject	Not reported
4D dynamic probabilistic atlas of developing brains (Kuklisova-Murgasova et al., 2011)	36.6 ± 4.9 weeks GA	142 (*M* = 70; *F* = 72)	• T2 • Tissue maps	• Voxel-wise weighted intensity averaging	Not reported
83 ROI 2-year old atlas (Gousias et al., 2008)	21.4–34.4 (24.8 ± 2.4) months	33 (*M* = 17; *F* = 16)	• T1 • T2 • ROI	• Single subjects	Not reported
A database of age-appropriate average MRI templates (Fillmore et al., 2015; Richards et al., 2016)	2 weeks–89 years^*^	2762	• T1 • T2 • Tissue maps	• Voxel-wise averaging	Reported
A multi-channel 4D probabilistic atlas of the developing fetal brain (Serag et al., 2012b)	29.6 ± 4.6 weeks GA	80	• T1 • T2 • Tissue maps	• Voxel-wise weighted intensity averaging	Not reported
A multi-modal map of human cerebral cortex (Glasser et al., 2016)	22–35 years	210 (*M* = 80; *F* = 130)	• T1 • T2 • tfMRI • rfMRI	• Group average parcellation	Not reported
A neonatal atlas template (Kazemi et al., 2007)	39–42 weeks GA	7 (*M* = 4; *F* = 3)	• T1	• Voxel-wise averaging	Not reported
A spatiotemporal atlas of MR intensity, tissue probability and shape of the fetal brain (Habas et al., 2010)	20.57–24.71 weeks GA	20	• SSFSE T2 • Tissue maps • ROI	• Voxel-wise averaging • Single subjects	Not reported
Adult brain maximum probability map: “Hammers adult atlases” (Hammers et al., 2003)	31.6 ± 9.9 years	30 (*M* = 15; *F* = 15)	• T1 • ROI	• Voxel-wise probabilities	Reported
Age-specific MRI templates for pediatric neuroimaging (Sanchez et al., 2012a)	4.5–24 years^*^	1289 (*M* = 636; *F* = 653)	• T1 • T2/PD	• Voxel-wise averaging	Reported
Allen Human Brain Atlas (Allen Institute for Brain Science, 2009)	24–57 years^*^ (post-mortem)	8 (*M* = 6; *F* = 2)	• T1 • T2	• Single subjects	Not reported
Automatic analysis of cerebral atrophy (Subsol et al., 1997)	37 years (mean)	10 (*M* = 10; *F* = 0)	• T1 • Ventricle map	• Average and SD feature positions	Reported
Bayesian interference atlases (Van Leemput, 2009)		18	• T1 • T2 • Tissue maps • ROI	• Bayesian inference averaging	Not reported
Brain atlas for healthy elderly^T&T^ (Lemaitre et al., 2005)	63–75 years	662 (*M* = 331; *F* = 331)	• T1 • Tissue maps	• Voxel-wise averaging	Reported
Brain Characterization Using Normalized Quantitative Magnetic Resonance Imaging (Warntjes et al., 2013)	26–67 (45 ± 11) years	31 (*M* = 14; *F* = 17)	• R_1_ • R_2_ • PD	• Voxel-wise averaging	Not reported
Brain Imaging of Normal Subjects (BRAINS) age-specific MRI atlases from young adults to the very elderly (Dickie et al., 2016a)	25–92 years^*^	225	• T1 • Tissue maps	• Voxel-wise averaging	Reported
Brain template for children from 2 weeks to 4 years age (Sanchez et al., 2012b)	8 days-4.4 years^*^	154 (*M* = 83; *F* = 71)	• T1 • T2/PD • ROI	• Voxel-wise averaging	Reported
Brainnetome atlas (Fan et al., 2016)	22–35 years	49 (*M* = 17; *F* = 32)	• T1 • T2 • Diffusion • rfMRI • ROI	• Voxel-wise probabilities	Not reported
Cerefy brain atlas^T&T^ (Nowinski, 2005)	60 years	1 (*M* = 0; *F* = 1)	• Digitised Talairach plates • ROI	• Single subject	Not reported
Chinese probabilistic atlas (Xing et al., 2013)	18–70 years^*^	1000	• T1 • T2 • Tissue maps	• Voxel-wise averaging	Reported
Chinese_56^T&T^ (Tang et al., 2010)	24.46 ± 1.81 years	56 (*M* = 56; *F* = 0)	• T1 • ROI	• Voxel-wise averaging	Reported
Clinical toolbox^T&T^ (Rorden et al., 2007)	72.9 ± 7.63years	50 (*M* = 18; *F* = 32)	• T1 • Tissue maps • CT	• Voxel-wise averaging	Not reported
Consistent high-definition spatio-temporal atlas of the developing brain (Serag et al., 2012a)	28–44 (37.3 ± 4.8) weeks PMA	204	• T1 • T2	• Voxel-wise averaging	Not reported
Construction of multi-region-multi-reference atlases (Shi et al., 2010)	1.3 ± 0.7 months	68 (*M* = 38; *F* = 30)	• T2 • Tissue maps • ROI	• Voxel-wise averaging	Not reported
Contributions to 3D Diffeomorphic Atlas Estimation: Application to Brain Images (Bossa et al., 2007)		19	• T1	• Voxel-wise averaging and SD	Not reported
Cortical gray matter of young adults (Luders et al., 2005)	25 ± 4 years	60 (*M* = 30; *F* = 30)	• T1 • Tissue maps • ROI	• Average and SD gyral locations	Not reported
Deformable Spatiotemporal MRI Atlas of the Fetal Brain (Gholipour et al., 2014)	26.14–35.86 (30.50 ± 3.05) weeks GA	40	• SSFSE	• Voxel-wise averaging	Not reported
Digital Pediatric Brain Structure Atlas (Shan et al., 2006)	9 years	1 (*M* = 0; *F* = 1)	• T1 • ROI	• Single subject	Reported
EvePM (Lim et al., 2013)	33 years	1 (*M* = 0; *F* = 1)	• T1 • Diffusion • ROI • susceptibility	• Single subject	Not reported
FreeSurfer “Destrieux” cortical atlas (Destrieux et al., 2010)	18–33 years	12 (*M* = 6; *F* = 6)	• T1 • ROI	• Vertex-wise probabilities	Not reported
Group-specific brain tissue probability map (Yoon et al., 2005)	26.07 ± 5.32 years	59 (*M* = 36; *F* = 23)	• T1 • Tissue maps • ROI	• Voxel-wise averaging	Reported
Harvard brain atlas (Shenton et al., 1995)	25 years	1 (*M* = 1; *F* = 0)	• T1 • ROI	• Single subject	Reported
Harvard-Oxford cortical and subcortical structural (Fmrib, 2008)	18–50 years	37 (*M* = 21; *F* = 16)	• T1 • ROI	• Voxel-wise probabilities	Not reported
Human cortical development map (Gogtay et al., 2004)	13.0 ± 4.8 years^*^	13 (*M* = 6; *F* = 7)	• T1 • GM map • ROI	• Average gyral locations	Reported
ICBM452^T&T^ (Lancaster et al., 2007)	20–40 years (27.8 ± 5.1) years	452	• T1 • T2 • Tissue maps • ROI	• Voxel-wise averaging	Not reported
Infant brain atlas (Altaye et al., 2008)	9–15 months	76 (*M* = 31; *F* = 45)	• T1 • Tissue maps	• Voxel-wise averaging	Not reported
Japanese pediatric standard brain (Uchiyama et al., 2013)	6–9 years	45 (*M* = 22; *F* = 23)	• T1	• Voxel-wise averaging	Reported
JHU-neonatal brain atlas (Oishi et al., 2011)	0–4 days	25 (*M* = 15; *F* = 10)	• T1 • T2 • Diffusion	• Voxel-wise averaging • Single subject	Not reported
Korean standard brain template (Lee et al., 2005)	18–77 (44.6 ± 19.4) years^*^	78 (*M* = 49; *F* = 29)	• T1 • F-18-FDG PET	• Voxel-wise averaging	Reported
LPBA40^T&T^ (Shattuck et al., 2008)	19–39 (29 ± 6) years	40 (*M* = 20; *F* = 20)	• T1 • Tissue maps • ROI	• Voxel-wise averaging • Voxel-wise probabilities	Reported
Merged young- and old-adult atlas target: “Washington 711”^T&T^ (Buckner et al., 2004)	49 years	24 (*M* = 9; *F* = 15)	• T1	• Voxel-wise averaging	Reported
Mindboggle-101 (Klein and Tourville, 2012)	19–61 years	101 (*M* = 57; *F* = 44)	T1 ROI	• Single subjects	Not reported
MNI/ICBM 152^T&T^ (Mazziotta et al., 2001)	18–44 (24 ± 7) years	152 (*M* = 86; *F* = 66)	• T1 • T2/PD • Tissue maps • ROI	• Voxel-wise averaging	Not reported
MNI 305^T&T^ (Evans et al., 1993)	23.4 ± 4.1 years	305 (*M* = 239; *F* = 66)	• T1 • Brain masks	• Voxel-wise averaging	Not reported
MNI Pediatric atlases^T&T^ (Fonov et al., 2011)	0–18.5 years^*^	324	• T1 • T2/PD • Tissue maps • Brain masks	• Voxel-wise averaging and SD	Not reported
MNI-Colin27^T&T^ (Holmes et al., 1998; Aubert-Broche et al., 2006)		1 (*M* = 1; *F* = 0)	• T1 • T2/PD • Tissue maps	• Voxel-wise averaging (of repeated single subject scans)	Not reported
Neonatal brain atlas: “ALBERT” (Gousias et al., 2012)	39–45 (41) weeks PMA	5 (*M* = 3; *F* = 2)	• T1 • T2 • ROI	• Single subjects	Reported
Neonatal brain template of 1 week newborn (Hashioka et al., 2012)	5.6 ± 17.6 days	14 (*M* = 11; *F* = 3)	• T2	• Voxel-wise averaging • Single subjects	Not reported
Neonatal probabilistic models (Kazemi et al., 2008)	39–42 weeks	7 (*M* = 3; *F* = 4)	• T1 • Tissue maps	• Voxel-wise averaging	Not reported
Non-parametric percentile rank atlas of the aging brain (Dickie et al., 2015a)	55–90 years	98 (*M* = 40; *F* = 58)	• T1 • GM map	• Voxel-wise non-parametric percentile ranking	Reported
Normal Brain F-18 FDG-PET and MRI Atlas (Schifter et al., 1993)		1	• T1 • T2 • FDG-PET	• Co-registration of within subject images	Not reported
Normal reference MR images for aging brain (Farrell et al., 2009)	65–80 years^*^	79 (*M* = 61; *F* = 18)	• T1 • T2	• Qualitative percentile ranking • Voxel-wise averaging	Reported
NTU standard Chinese brain template (Jao et al., 2009)	19–42 (25.7) years	95 (*M* = 50; *F* = 45)	• T1	• Voxel-wise averaging	Reported
Parcellation of the Healthy Neonatal Brain into 107 Regions (Blesa et al., 2016)	39–47^+1^ (42^+2^) weeks	33	• T1 • T2 • Diffusion • Tissue maps • ROI	• Voxel-wise majority voting	Reported
Population difference in brain among Chinese, Malay and Indian neonates (Bai et al., 2012)	5–17 days	177 (*M* = 94; *F* = 83)	• T2 • Diffusion	• Voxel-wise averaging	Reported
Population-Average, Landmark- and Surface-based (PALS) atlas (Van Essen, 2005)	18–24 years	12 (*M* = 6; *F* = 6)	• T1 • Cortical surface	• Selected landmark averaging	Not reported
Regional growth and atlasing of the developing human brain (Makropoulos et al., 2016)	39^+1^ (27^+1^–44^+6^) weeks PMA	338	• T1 • T2 • Tissue maps • ROI	• Voxel-wise averaging	Not reported
Resource atlases for multi-atlas brain segmentations with multiple ontology levels based on T1-weighted MRI (Wu et al., 2016)	4–82 years^*^	90	• T1 • ROI	• Hierarchical ontology	Not reported
Spatial–temporal fetal atlas (Zhan et al., 2013)	15–22 weeks GA^*^	34 (*M* = 12; *F* = 22)	• T2	• Voxel-wise averaging and SD	Reported
SRI24 (Rohlfing et al., 2010)	19–84 (52 ± 5) years	24 (*M* = 12; *F* = 12)	• T1 • T2/PD • Diffusion • Tissue maps • ROI	• Voxel-wise averaging	Reported
Symmetric atlas in normal older adults^T&T^ (Grabner et al., 2006)	75 ± 6 years	153	• T1 • ROI	• Voxel-wise averaging	Not reported
Talairach and Tournoux^T&T^ (Talairach and Tournoux, 1988; Brett et al., 2001)	60 years	1 (*M* = 0; *F* = 1)	• Histological slices • Photographs • Hand drawings • Stereotactic coordinates	• Postmortem slicing • Photography • Drawing	Not reported
The human brain in 1700 pieces (Nowinski et al., 2012)		1 (*M* = 0; *F* = 1)	• T1 • 3D TOF • SWI • Diffusion • ROI	• Single subject	Not reported
The pediatric template of brain perfusion (Avants et al., 2015)	7–18 years	120 (*M* = 59; *F* = 61)	• T1 • BOLD • Diffusion • pCASL • ROI • Tissue maps	• Voxel-wise averaging	Reported
Three-dimensional digitized mono-subject anatomical template (Lalys et al., 2010)	45 years	1 (*M* = 1; *F* = 0)	• T1 • T2	• Voxel-wise kappa-sigma clipping average (of repeated single subject scans)	Not reported
UNC Infant 0–1–2 atlases (Shi et al., 2011)	0–2 years	95 (*M* = 56; *F* = 39)	• T1 • T2 • Tissue maps • ROI	• Voxel-wise averaging • Voxel-wise majority voting (maximum probability)	Reported

*Empty or partially empty cells indicate that we could not find relevant data in original manuscripts*;

* *age-specific atlases generated within age range*;

a *age is reported as in the original manuscript and is shown “range (mean ± SD)” if available; MRI, magnetic resonance imaging; SD, standard deviation; ROI, region of interest; PD, proton density; SWI, susceptibility weighted imaging; tfMRI, task-based functional magnetic resonance imaging; rfMRI, resting-state functional magnetic resonance imaging; PMA, post-menstrual age; GA, gestational age; pCASL, pseudo continuous arterial spin labeled; BOLD, blood oxygen level-dependent; SSFSE, single shot fast spin echo; M, male; F, female; T&T, developed by or descended from Talairach and Tournoux*.

We found 66 structural brain MRI atlases with a total of 10,354 subjects (median = 43, mean = 157, range = 1–2762), including European, North American, Chinese, Japanese, Korean, Indian, and Malay participants.

We identified 19 fetal, neonate and infant (0–5 years); six childhood (5–18 years); 23 young or middle aged adult (18–60 years); seven older adult (aged >60 years); and six life-course atlases including several age groups. Five atlases did not report the age of included subjects.

Twenty-seven atlases (41%) reported cognitive/clinical data but this was generally in summary form, e.g., “subjects had no history of neurological, psychiatric or other significant medical illnesses” (Lee et al., 2005) rather than summarized measures from individual subjects. One atlas of the elderly brain reported data on age, handedness, MMSE, education level, and proportion of hypertensive subjects (Lemaitre et al., 2005), but we found no atlas that reported a comprehensive battery of cognitive, medical, and demographic data that are increasingly found in large cohort studies (Wardlaw et al., 2011; Deary et al., 2012).

All atlases were based on T1, T2, and/or PD structural sequences. No atlas included FLAIR or T2^*^ sequences. Almost all multiple subject atlases (except Farrell et al., 2009; Dickie et al., 2015a); were derived using parametric mean-based methods rather than non-parametric percentile ranks or ranges.

Some atlases used the same publicly available databases, e.g., Open Access Series of Imaging Studies (OASIS) data were used in at least two atlases (Dickie et al., 2015a; Richards et al., 2016). We were not able to quantify the subject overlap between atlases as subject identifiers were generally not provided. Ten atlases were based on a single subject. We identified 13 atlases (19.7%) that were developed by or descended from Talairach and Tournoux (labeled “T&T” in Table 1).

## Discussion

Brain atlases are an important resource for neuroanatomical definition and are often the basis for automated image analyses, which are likely to become increasingly used for population imaging studies. It is important that users are aware of the origins and assumptions underlying these atlases. We identified 66 whole brain structural MRI atlases with a total of 10,354 “normal” subjects from 15 weeks gestational age to 92 years. The number of subjects in each atlas was generally rather small (median = 43; mean = 157; range = 1–2762; *n* ≥ 100 = 18; *n* ≥ 1000 = 3) given that several hundreds or even thousands of subjects are required to represent population brain structure adequately (Mazziotta et al., 2001; Toga, 2002; Toga et al., 2006; Evans et al., 2012). Only 622 subjects (6%) had measures of medical, cognitive, and demographic data to support their classification as normal (Lemaitre et al., 2005). Thirteen atlases (~20%) were descended from the Talairach and Tournoux atlas (Talairach and Tournoux, 1988), e.g., MNI, ICBM, and “Brain atlas for healthy elderly.”

Specific populations should be analyzed using an atlas derived from other subjects in that population, or a closely relevant population, otherwise systematic errors may be introduced, e.g., the overexpansion of atrophied brains registered to younger subject atlases (Buckner et al., 2004). Relevant to this, we suggest that the most appropriate atlas for a given study (should there be multiple atlases available with similar demographic, clinical, and cognitive profiles) is the one which requires the least amount of global or regional warping from native subject space to atlas space (and vice-versa). The consequences of various degrees of processing and warping individual subjects to an atlas space have previously been analyzed and discussed (Dickie et al., 2015a). The presence of cognitive deficits and medical conditions, e.g., vascular risk factors, also affect brain structure (Ritchie et al., 2015; Dickie et al., 2016b) and therefore it is essential for this information to be measured and tabulated in brain atlases. Although we appreciate that such depths of data may be difficult and expensive to acquire their strong influence on brain structure makes them imperative for understanding the appearance and structure of brain atlases. Medical, cognitive, and demographic data that may be useful in understanding the structure of atlases at different stages of life have been described previously (Job et al., 2016). Given the wide variation and features of brain structure across the life course (Good et al., 2001; Sowell et al., 2003; Allen et al., 2005; Raz et al., 2010), reliable studies, particularly at the extremes of life, require atlases with many more subjects including clinical and cognitive data and additional structural MRI sequences, e.g., T2-based sequences for measuring burden of small vessel disease (Wardlaw et al., 2013).

Such “big-data” approaches including a wide number of imaging sequences and supporting textual information have been successfully applied in studies with limited age ranges such as the “Human Connectome Project” which aims to map structural and functional connections in the healthy brain between ages 22 to 35 years (Van Essen et al., 2012) and UK Biobank (Miller et al., 2016). The challenge is to collect similarly rich and relevant data, including sequences such as T2^*^ and FLAIR and vascular risk factor measures for appropriately characterizing cerebrovascular and cognitive development/aging effects on brain structure, at the extremes of life. An international collaborative and aggregative approach may be the best way of achieving this goal as was recently agreed by a panel of experts in structural brain mapping in 2014 (Job et al., 2016) and as is evidenced in similar efforts in functional imaging (Zuo et al., 2014). Although there are challenges to aggregating brain MRI from multiple centers/scanners, particularly in functional connectomics (Zuo and Xing, 2014), these issues have received great attention (e.g., Gountouna et al., 2010; Gradin et al., 2010) and the variability between scanners has often shown to be nominal compared to the great variability in brain structure among even people of the same age, gender, and cognitive status (Dickie et al., 2013; Ritchie et al., 2015; Miller et al., 2016).

High resolution structural MRI is increasingly used in population imaging to study brain development in fetal (pre-birth), neonatal (birth to 4 weeks corrected gestational age) and pediatric (1 month to 18 years) populations because of its utility to: provide quantitative measures of typical brain growth; map atypical growth following complications such as preterm birth, perinatal asphyxia and stroke; evaluate tissue effects of neuroprotective treatment strategies; identify the neural substrates of long-term neurodevelopmental impairments; and because it has potential to uncover early life origins of adult neurological and psychiatric disease. All of these applications benefit from the anatomic context provided by atlases.

There are challenges in analyzing structural images in early and late life. These begin during image acquisition and extend into image analysis. For example, infant participants are asleep during scanning while adults are usually awake; motion artifacts are generally low in mid-life but increase at the extremes of life; and heart and respiratory rates also vary greatly through life (Zuo et al., 2017). Brain structural patterns also very greatly though life: in early life growth is rapid and head shape and size varies, with a changes in tissue composition and relatively low spatial resolution (Matsuzawa et al., 2001). In older people there is accelerated brain tissue loss, reduced cortical contrast, white matter disease, enlarged perivascular spaces, stroke infarcts, and microbleeds, among other features (Raz et al., 2010; Wardlaw et al., 2013; Dickie et al., 2016b). There have been several (*N* = 19) fetal, neonate, or infant (<age 5) atlases published, but our review found relatively limited age-specific childhood (*N* = 6: >5 and <18 years) and older adult atlases (*N* = 7: >60 years) compared to young/middle-aged adult atlases (*N* = 23). Despite their current under-representation in the literature, age-specific atlases in childhood, and old age may have important uses in research and clinical practice, such as providing targets for aiding classification and diagnoses of developmental and neurodegenerative diseases (Farrell et al., 2009; Dickie et al., 2013, 2014), particularly since better understanding of normal development, aging, and dementia prevention are major focuses of many large population studies.

Most atlases we found were based on mean/parametric statistics and designed to provide a standard space for voxel-wise analyses or support tissue/ROI volume segmentation. In contrast, the “Normal reference MR images for the brain” atlas was based on qualitatively determined percentile ranks of brain volumes during normal aging and designed to support clinical diagnoses of whole brain volume loss in aging (65–70 and 75–80 year old) patients (Farrell et al., 2009). These clinical atlases are designed to “calibrate” differences in perception between neuroradiologists and have been of growing interest and in increased use since their inception in 2009 (Farrell et al., 2009; Hoggard, 2009; Job et al., 2016). Additionally, increased interest in use of computational automated image processing in clinical practice, e.g., to assess brain, hippocampus, or white matter lesion volumes, relies on availability of relevant and reliable age-relevant atlases. Atlases based on parametric statistics, e.g., mean and standard deviation, are not suitable to define the irregular brain volume distributions in old age (Dickie et al., 2013, 2015a). Therefore, non-parametric statistics were recently applied quantitatively to derive voxel-based percentile ranks and limits of normal aging GM, but this atlas was limited by the use of only T1 sequences and a wide age range (Dickie et al., 2015a). Further, work in developing non-parametric distributional representations of the brain, including a broad range of sequences in well-described (cognitively and medically) age-specific groups, may lead to clinically useful atlases for supporting diagnoses of developmental and neurodegenerative disease (Farrell et al., 2009; Wardlaw et al., 2013; Dickie et al., 2014).

The strengths of our review include the use of structured methods, that were reported following the PRISMA Guidelines (Moher et al., 2009), over ~6 years. We also conducted an exhaustive manual search of printed and online materials, and provided a structured evaluation of brain atlases according to pre-specified criteria. This allowed us to produce a holistic review of structural MRI brain atlases from across the life course in detail that we have not found previously. But despite these strengths, our review also has some limitations. The atlases we found were openly published, and identified through a formal search thus we may not have identified all relevant atlases, e.g., those described as part of larger studies (and therefore potentially not visible through traditional search methods) or those not published/openly accessible. We report data as described in the paper or website, and it is possible that additional data, e.g., on subjects' age, sex, clinical information, was collected and may have been published elsewhere. We did not contact authors for additional information. Further, we did not investigate potential uses for atlases beyond those described in the original manuscripts/sources. It could be that any one of these atlases may be modified to serve additional purposes. Related to this, we described the methods and uses of each atlas according to our interpretation of the source manuscripts/reference manuals, which may differ from the meaning intended by the original authors.

Notwithstanding these limitations, we have reviewed and described structural MRI brain atlases from across the life course and found that they were mostly of modest size with limited supporting subject information, developed with restricted image sequences for specific processing purposes, and that childhood and elderly populations were under-represented. We conclude that there is a continuing need for multi-sequence structural MRI, and the associated clinical, medical, and demographic data, collected in population imaging studies to be made widely available (with appropriate legal and ethical approvals) to create non-parametric brain atlases that adequately reflect the variability and features of brain changes throughout the life course. Brain image databanks, such as Brain Imaging in Normal Subjects (BRAINS; https://www.brainsimagebank.ac.uk/; Job et al., 2016), should work together to maximize sample sizes, generalizability and optimize data use to benefit analyses in population imaging studies and in future clinical practice.

## Author contributions

DAD and JL conducted systematic searches of the literature and internet. DAD, SS, JL, DA, MBC, and JB, conducted hand searching and reviewing of the literature and internet. DAD and SS wrote the manuscript. DAD, SS, JL, DA, MBC, JB, AW, DR, DJ, and JW edited the manuscript. DAD, SS, DR, DJ, and JW conceptualized and designed the study.

### Conflict of interest statement

The authors declare that the research was conducted in the absence of any commercial or financial relationships that could be construed as a potential conflict of interest.
